# Amphiregulin regulates odontogenic differentiation of dental pulp stem cells by activation of mitogen-activated protein kinase and the phosphatidylinositol 3-kinase signaling pathways

**DOI:** 10.1186/s13287-022-02971-4

**Published:** 2022-07-15

**Authors:** Junqing Li, Zhihua Wang, Juan Wang, Qian Guo, Yi Fu, Zihan Dai, Minghao Wang, Yu Bai, Xin Liu, Paul R. Cooper, Jiayuan Wu, Wenxi He

**Affiliations:** 1 State Key Laboratory of Military Stomatology & National Clinical Research Center for Oral Diseases, Shaanxi Key Laboratory of Stomatology, Department of Operative Dentistry & Endodontics, School of Stomatology, The Fourth Military Medical University, 145 Chang-le Road, Xi’an, 710032 People’s Republic of China; 2 Department of Oral Sciences, Sir John Walsh Research Institute, Faculty of Dentistry, University of Otago, Te Whare Wānanga O Otāgo, PO Box 56, Dunedin, 9054 New Zealand; 3 Hospital of Stomatology, Zunyi Medical University, 89 Wu-jiang Dong Road, Zunyi, 563003 People’s Republic of China; 4 Department of Stomatology, Air Force Medical Center, Air Force Medical University, 30 Fucheng Road, Beijing, 100142 People’s Republic of China

**Keywords:** Cell differentiation, Signal transduction, Tissue regeneration

## Abstract

**Background:**

Human dental pulp stem cells (hDPSCs) have received widespread attention in the fields of tissue engineering and regenerative medicine. Although amphiregulin (AREG) has been shown to play a vital function in the biological processes of various cell types, its effects on DPSCs remain largely unknown. The aim of this study was to explore the specific role of AREG as a biologically active factor in the regeneration of dental pulp tissue.

**Methods:**

The growth of hDPSCs, together with their proliferation and apoptosis, in response to AREG was examined by CCK-8 assay and flow cytometry. We explored the effects of AREG on osteo/odontogenic differentiation in vitro and investigated the regeneration and mineralization of hDPSCs in response to AREG in vivo. The effects of AREG gain- and loss-of-function on DPSC differentiation were investigated following transfection using overexpression plasmids and shRNA, respectively. The involvement of the mitogen-activated protein kinase (MAPK) or phosphatidylinositol 3-kinase (PI3K)/Akt pathways in the mineralization process and the expression of odontoblastic marker proteins after AREG induction were investigated by using Alizarin Red S staining and Western blotting, respectively.

**Results:**

AREG (0.01–0.1 µg/mL) treatment of hDPSCs from 1 to 7 days increased hDPSCs growth and affected apoptosis minimally compared with negative controls. AREG exposure significantly promoted hDPSC differentiation, shown by increased mineralized nodule formation and the expression of odontoblastic marker protein expression. In vivo micro-CT imaging and quantitative analysis showed significantly greater formation of highly mineralized tissue in the 0.1 μg/mL AREG exposure group in DPSC/NF-gelatin-scaffold composites. AREG also promoted extracellular matrix production, with collagen fiber, mineralized matrix, and calcium salt deposition on the composites, as shown by H&E, Masson, and Von Kossa staining. Furthermore, AREG overexpression boosted hDPSC differentiation while AREG silencing inhibited it. During the differentiation of hDPSCs, AREG treatment led to phosphorylation of extracellular signal-regulated kinase (ERK), c-Jun N-terminal kinase (JNK), and PI3K/Akt. Notably, a specific inhibitor of ERK, JNK, and PI3K/Akt signaling markedly reduced AREG-induced differentiation, as well as levels of phosphorylated ERK and JNK in hDPSCs.

**Conclusions:**

The data indicated that AREG promoted odontoblastic differentiation and facilitated regeneration and mineralization processes in hDPSCs.

**Supplementary Information:**

The online version contains supplementary material available at 10.1186/s13287-022-02971-4.

## Background

Mesenchymal stem cells (MSCs) are multifunctional cells present in various tissues, including bone marrow, adipose tissue, Wharton’s jelly, umbilical cord blood, and peripheral blood, that can be used for cell therapy and tissue/organ regeneration [1, 2]. Dental pulp stem cells (DPSCs) isolated from human dental pulp are specific mesenchymal stem cells that can be induced to differentiate into odontoblasts like cells in vitro or to form dentine-/pulp-like tissue in vivo [3–6]. DPSCs are relatively easy to obtain from waste tooth tissue and exhibit relatively high levels of proliferation, clonal potential, and mineralization compared with bone marrow mesenchymal stem cells (BMMSCs) [7]. Consequently, DPSCs have received widespread attention in the fields of tissue engineering and regenerative medicine. Despite this, signal transduction mechanisms involved in the oriented differentiation of DPSC are not yet fully understood.

Growth factor signaling plays a pivotal regulatory role in both cell growth and differentiation. Amphiregulin (AREG) is a member of the epidermal growth factor (EGF) family and is expressed by a variety of epithelial and mesenchymal cell types during development; it is also associated with homeostasis [8, 9]. According to previous reports, AREG is transcribed as a 1.4-kb mRNA composed of six exons that encode for a transmembrane polarized glycoprotein precursor (Pro-AREG) comprised of 252 aminoacids [8]. As a membrane-anchored precursor protein, it is mainly engaged in juxtacrine signaling on adjacent cells. There are three different levels of regulation of AREG functionality and these include: (1) at the transcriptional level through the binding of activators and repressors to different regions on the AREG promoter, (2) at the post-transcriptional level through modulation of mRNA stability, and (3) at the post-translational level through covalent modifications and the regulation of AREG shedding [8]. Recently, AREG has been implicated in the regulation of a wide range of biological processes, including cell growth, proliferation, neurogenesis, cell migration, and bone formation, by binding to the EGF receptor (EGFR) on the cell membrane [10]. Interestingly, AREG can regulate squamous cell differentiation and neuronal differentiation from stem/progenitor cell sources [11, 12]. AREG has also been found to accumulate in multiple myeloma-derived exosomes and is involved in regulating osteoclast differentiation through a circuitous mechanism in osteoblasts [13]. However, despite these demonstrations of AREG’s role in the differentiation of a variety of cell types, its effects on the growth and differentiation of DPSCs remain largely unknown.

AREG was initially identified in culture supernatants from the human breast cancer cell line MCF-7 [14] and was found to bind to EGFR. Subsequently, it has been shown to activate a variety of downstream intracellular signaling pathways, including Ras/MAPK, PI3K/AKT, mTOR, and STAT [15, 16]. These signal transduction cascades regulate gene expression and initiate diverse cellular responses, including proliferation, survival, invasion, differentiation, and angiogenesis. AREG can promote migratory activity and doxorubicin resistance through activation of the MAPK pathway [17]. Furthermore, AREG-induced growth can also be regulated partially through the MAPK and PI3K-Akt/PKB pathways [18]. Currently, the role of AREG in activating downstream signaling in the regeneration of dental pulp tissue is not known. Therefore, our study aimed to explore the effects of AREG on the growth, differentiation, and regeneration processes of DPSCs.

## Methods

### Reagents

Amphiregulin (AREG) (262-AR-100) was obtained from R&D Systems (R&D, CA, USA). The specific inhibitors of ERK kinase, p38 kinase, JNK kinase, and AKT kinase are U0126, SB203580, SP600125, and LY294002, respectively, were all obtained from Cell Signaling Technology (Danvers, MA, USA). AREG lentivirus kits were purchased from Hanbio Biotechnology (Hanbio, China).

### DPSC culture

Freshly extracted sound teeth were collected from patients (18–22-years-old), under the approval of the Ethics Committee of the Fourth Military Medical University (FMMU), Xi’an, China with informed consent. The hDPSCs were isolated, cultured, and characterized as previously described [19, 20]. Briefly, the pulp tissue was dissected and digested with 3 mg/mL type I collagenase and 4 mg/mL dispase (Sigma-Aldrich, St Louis, MO, USA) for 45–60 min at 37℃. Single-cell suspensions were cultured in 60-mm culture dishes and maintained in a-minimum essential medium (α-MEM; Invitrogen, Carlsbad, CA, USA) with 10% (v/v) fetal bovine serum (Gibco-BRL, Grand Island, NY, USA), 100 units/mL penicillin-G, and 100 mg/mL streptomycin (Invitrogen) in a humidified atmosphere with 5% CO_2_ at 37℃. Single-cell clones of DPSCs were isolated and passaged as previously described [21]. DPSCs were grown in a 5% CO_2_ incubator at 37 °C, and cultures at passages 3–5 were used for all studies.

### Multi-lineage differentiation in vitro

Cells were cultured in 6-well plates in osteo/odontogenic induction medium containing 50 mg/mL ascorbic acid, 10 mM β-glycerophosphate, and 10 nM dexamethasone (all from Sigma-Aldrich) with 10% FBS for 14 days. The cells were washed twice in PBS and fixed with 4% polyoxymethylene (Sigma-Aldrich, St Louis, MO, USA) for 15 min. The cells were then stained with Alizarin Red S (Sigma-Aldrich, St Louis, MO, USA). For adipogenic induction, the cells were cultured in adipogenic induction medium containing 1 mM IBMX, 0.4 mM indomethacin, 0.2 μM dexamethasone, 0.02 mg/mL insulin, 100 μg/mL streptomycin, 100 U/mL penicillin, and 10% FBS in α-MEM medium) for 21 days. Cultures were stained with 0.3% (w/v) Oil-Red O (Sigma-Aldrich)/60% isopropanol reagent for 60 min. Finally, cultures were washed three times in water prior to analysis.

### Flow cytometric analysis of stem cell surface markers

DPSCs (2 × 10^5^ cells) were digested by trypsinization and harvested. Cells (1 × 10^6^/100 µL) were resuspended and incubated with fluorescein isothiocyanate (FITC)-coupled anti-human monoclonal antibodies against CD105-FITC, CD34-PE, CD45-PE, CD90-PE, CD29-PE, and CD146-PE (1: 100 dilution; BD Biosciences, San Jose, CA, USA) in PBS with 3% FBS for 1 h in the dark at room temperature. Finally, the samples were analyzed on a flow cytometer (FACS Calibur; BD Biosciences, Franklin Lakes, NJ, USA) using CellQuest PROTM software (BD Biosciences).

### Flow cytometric analysis of proliferation and apoptosis in hDPSCs

Third-passage DPSCs (3 × 10^6^ cells/dish) were inoculated in 60 mm dishes. The experimental groups were treated with 0.01 μg/mL and 0.1 μg/mL AREG for 16 h, respectively. The control group received no AREG treatment. After removal from the dishes, cells were washed three times in pre-cooled phosphate-buffered saline (PBS) and were fixed in 75% ice-cold ethanol. A flow cytometer (BD Biosciences, San Jose, CA, USA) was used to monitor the changes in the G0/G1, S, and G2/M phases of the cell cycle and to calculate the cell proliferation index (PI = G2/M + S). For the measurement of apoptosis, the AREG-treated DPSCs were harvested and washed as above, and 500 μl of cells was diluted with 1 × Annexin V Binding Buffer working solution according to the instructions of the apoptosis kit (KeyGENBioTECH, China). Then, 5 μl Annexin V-APC and 5μL 7-ADD staining solution were added to the cells and the number of apoptotic cells was detected by FCM.

### Cell counting kit-8 assay (CCK-8)

Five thousand DPSCs per well were seeded and cultured in 96-well plates with a range of concentrations of AREG (0-1 μg/mL). In a separate experiment, DPSCs were incubated with 20 µL CCK-8 reagents (KeyGENBioTECH, China) at 37 °C for 2 h for 1, 3, 5, and 7 days. Finally, absorbances at 450 nm were read in a microplate reader (ModelELX 808; Bio-Tek, Winooski, VT, USA).

### Alizarin Red S staining and quantitation

DPSCs (4 × 10^5^ cells/well) were seeded into 6-well plates and cultured in either control medium or osteo/odontogenic induction medium with AREG for 14 days. At specified times, the cells were fixed in 1 ml of 4% paraformaldehyde for 30 min at room temperature. After three washes with distilled water, 0.1 g/ml Alizarin Red S (ARS) (Sigma-Aldrich) was used to stain the cultures for 10 min at room temperature. The unbound stain was removed by washing with deionized water until the discarded liquid appeared colorless. Five hundred microliters of water were added to each dish to ensure the cells remained hydrated. Cells were observed under an Olympus inverted microscope (Tokyo, Japan). For quantitation, alizarin red S stain dissolved in 10% cetylpyridinium chloride (CPC) (Sigma-Aldrich) was added to the cells and the absorbance at 450 nm was read in the microplate reader.

### In vivo studies

All animal surgical procedures were approved by the Animal Care Committee and the Institutional Review Board (IRB) for Human Subjects Research of the Fourth Military Medical University. The high-stiffness three-dimensional (3D) nanofibrous gelatin (NF-gelatin) scaffolds were a kind gift from Prof. Tiejun Qu [22]. Initially, the NF-gelatin scaffolds were placed in 70% alcohol for half an hour and then washed three times in sterile PBS to remove residual ethanol. Before the seeding of human DPSCs (5 × 10^5^), the scaffolds were soaked in α-MEM containing 10% FBS. The cell-scaffold composites were cultured in α-MEM supplemented with 10% FBS for 24 h on an orbital shaker (Orbi-shaker™, Benchmark, USA) in an incubator with 5% CO_2_ at 37℃. Subsequently, the cell-scaffold composites were stimulated with 0.1 μg/mL AREG in osteo/odontogenic induction medium for 7 days. Controls received no AREG exposure. The medium was changed every other day. After 7 days, the cell-scaffold composites were implanted subcutaneously on the dorsal surfaces of immune-compromised nude mice (nu/nu, 6–8 weeks old). After 4 weeks, the mice were euthanized by an anesthetic overdose and the tissue growths were then harvested. Tissue samples were immediately fixed in 4% paraformaldehyde overnight. The samples (*n* = 4 in each group) were scanned and analyzed using a micro-CT (eXplore Locus SP micro-CT; GE Healthcare, USA) as previously described [23] and the 3D micro-architectural properties of specimens were evaluated using analysis software (MicroView; GE Healthcare). After decalcification in 17% ethylenediamine tetra-acetic acid, hematoxylin–eosin (H&E), Masson’s trichrome, and von Kossa staining were used for histological observation.

### Overexpression and knockdown of AREG in DPSCs

AREG-green fluorescent protein lentivirus kits and their respective control kits were purchased from Hanbio (China). DPSCs were transfected with AREG lentivirus according to the manufacturer's instructions. In brief, third-passage DPSCs were inoculated in 12-well plates at 0.5 × 10^5^/mL and infected for 4 h, after which 0.5 mL fresh complete medium was added. After a further 24-h infection period, the medium was replaced with 1 mL fresh medium and after a further 72 h, the transfection rate was examined under a fluorescence microscope (DMI8, Leica, German). Puromycin was used to select stably transfected cells with the puromycin concentration determined in a preliminary experiment. For simplicity, *AREG*-overexpressing and *AREG*-silenced cells were referred to as AREG (+) and AREG (−), respectively. The effects on the oriented differentiation of DPSCs were examined by ARS and Western blot analysis as above and described above and below.

### Western blot analysis

DPSCs were cultured in serum-free medium for 24 h and were treated with 0.1 µg/mL AREG for 0, 30, 60, and 90 min in the presence or absence of the specific inhibitors U0126 (25 µM), SP600125 (25 μM), SB203580 (25 μM), and LY294002 (10 μM). Protein extraction from the cells, SDS-PAGE, and blotting was performed as previously described [17]. After incubating with secondary antibody (1:4000; Santa Cruz Biotechnology, Dallas, TX, USA) for 1 h, the protein bands were imaged by using an enhanced chemiluminescence system (Amersham, Piscataway, NJ, USA).

### Statistical analysis

All experiments were repeated separately in triplicate or quintuplicate. Data are expressed as means ± SD. For statistical processing, we used SPSS software 16.0 (version 16.0; SPSS, Chicago, IL, USA). Inter-group differences were compared by the ONE test. *P* < 0.05 was considered to be statistically significant.

## Results

### Culture and characterization of hDPSCs

**Fig. 1:**
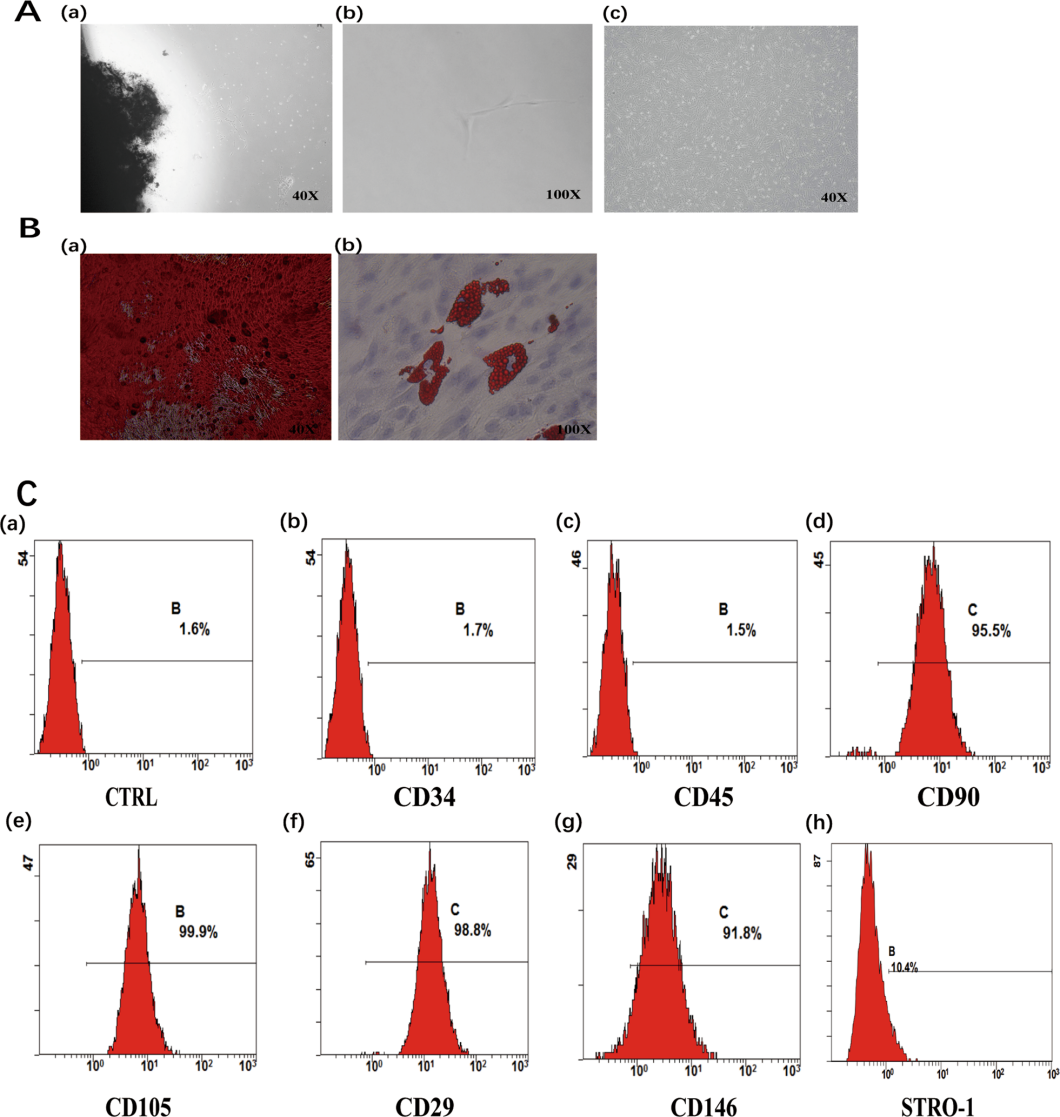
Culture and characterization of DPSCs. **A** (**a**) DPSCs isolated and cultured from pulp tissue samples, (**b**) clonal DPSCs, (**c**) were cultured for 2 weeks. **B** (**a**) Mineralized nodule formation in osteo/odontogenic induction medium demonstrated by Alizarin Red S staining, (**b**) lipid droplet formation in adipogenic induction medium using Oil Red O staining. **C** Flow cytometry of molecular surface antigen markers in DPSCs. (**a**) Negative control for DPSCs. Representative profiles are shown for the expression of the immunophenotypic markers CD34 (**b**), CD45 (**c**), CD90 (**d**), CD105 (**e**), CD29 (**f**), CD146 (**g**) and STRO-1 (**h**) in DPSCs

Fibroblast-like clonal cells were obtained from the dental pulp tissue by limiting dilution and colony cloning (Fig. 1A a, b). Putative stem cells derived from the clonal cells (Fig. 1A c) were characterized by multiple lineage differentiation tests and flow cytometry. If cultured under inductive conditions, the cells formed mineral nodules and lipid droplets, shown by Alizarin Red S and Oil Red O staining (Fig. 1B a, b). Flow cytometry analysis showed that CD90, CD105, CD29, CD146, and STRO-1 were highly expressed in isolated cultured DPSCs (Fig. 1C d–h). In contrast, the hematopoietic cell markers CD34 and CD45 (Fig. 1C b, c) were detected at minimal levels in the cultured stem cells. Analysis of morphology, colony formation, immunophenotype, and the ability to differentiate into multiple lineages indicated that mesenchymal stem cells had been isolated.

### Effects of AREG on growth of hDPSCs

**Fig. 2:**
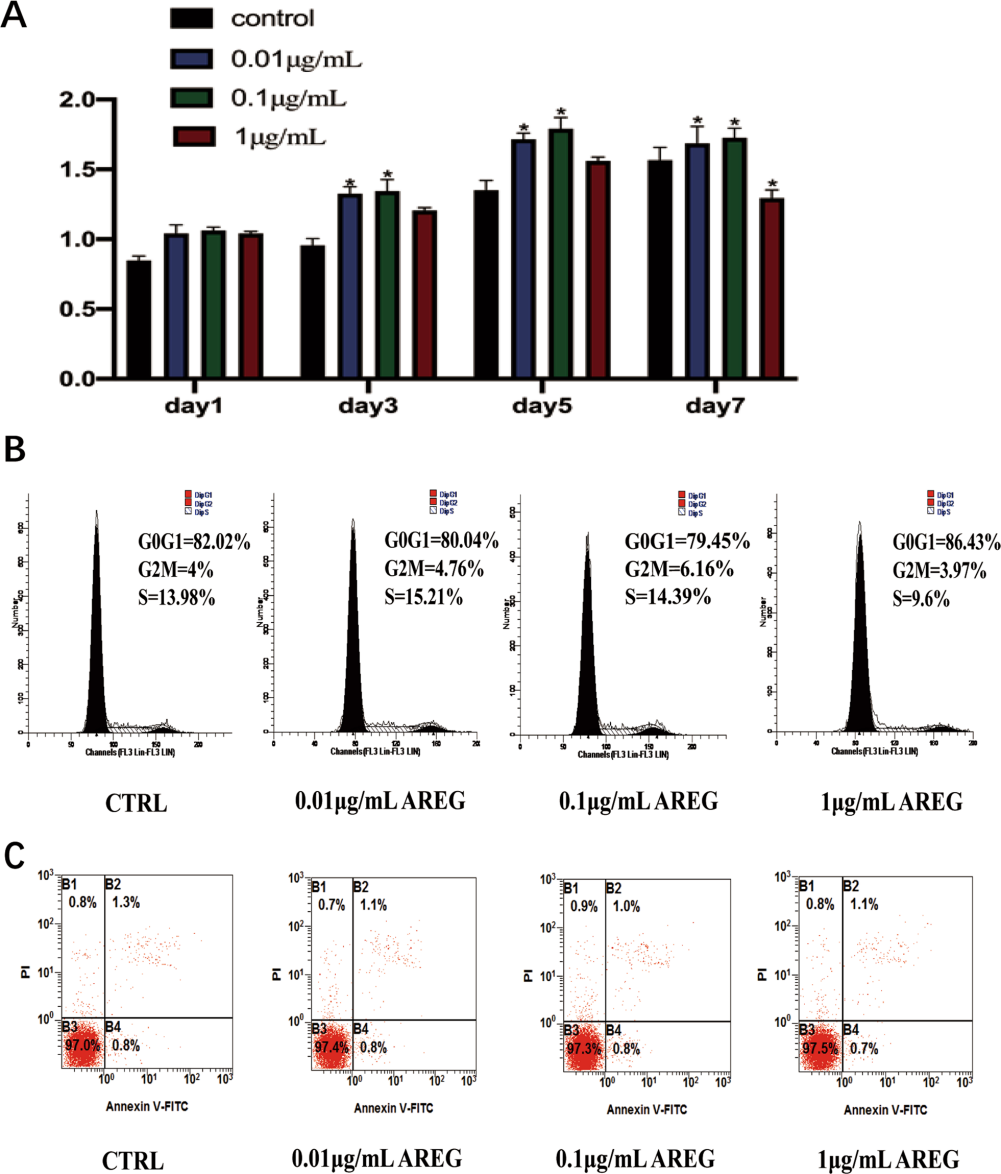
Effects of AREG on the growth of DPSCs. **A** The CCK-8 assay was used to detect cell proliferation in different AREG-treated groups (0.01–1 mg/mL) and untreated DPSCs at 1, 3, 5 and 7 days (*n* = 5, *P < 0.05). **B** The cell cycle proliferation index (PI = G2/M + S) in AREG-treated and control groups analyzed by flow cytometry. **C** Flow cytometry analysis of AREG-treated and control groups

The effects of AREG on hDPSC numbers after 1-, 3-, 5-, and 7-days incubation were assessed using the CCK-8 assay (Fig. 2A). The data showed that cell numbers in the 0.01 μg/mL and 0.1 μg/mL AREG-treated groups were significantly increased (*P* < 0.05). In contrast, cell numbers in the 1 μg/mL AREG-treated group were reduced. Cell cycle analysis showed a minimal increase in the proliferation index in the 0.01 μg/mL and 0.1 μg/mL AREG exposure groups (PI = G2/M + S), whereas treatment with 1 μg/mL AREG decreased the proliferation index compared with the control group (CTRL) (Fig. 2B). In addition, FCM analysis showed that AREG did not significantly affect apoptosis of DPSCs compared with the CTRL group (Fig. 2C).

### Effects of AREG on odontogenic differentiation of hDPSCs in vitro

**Fig. 3:**
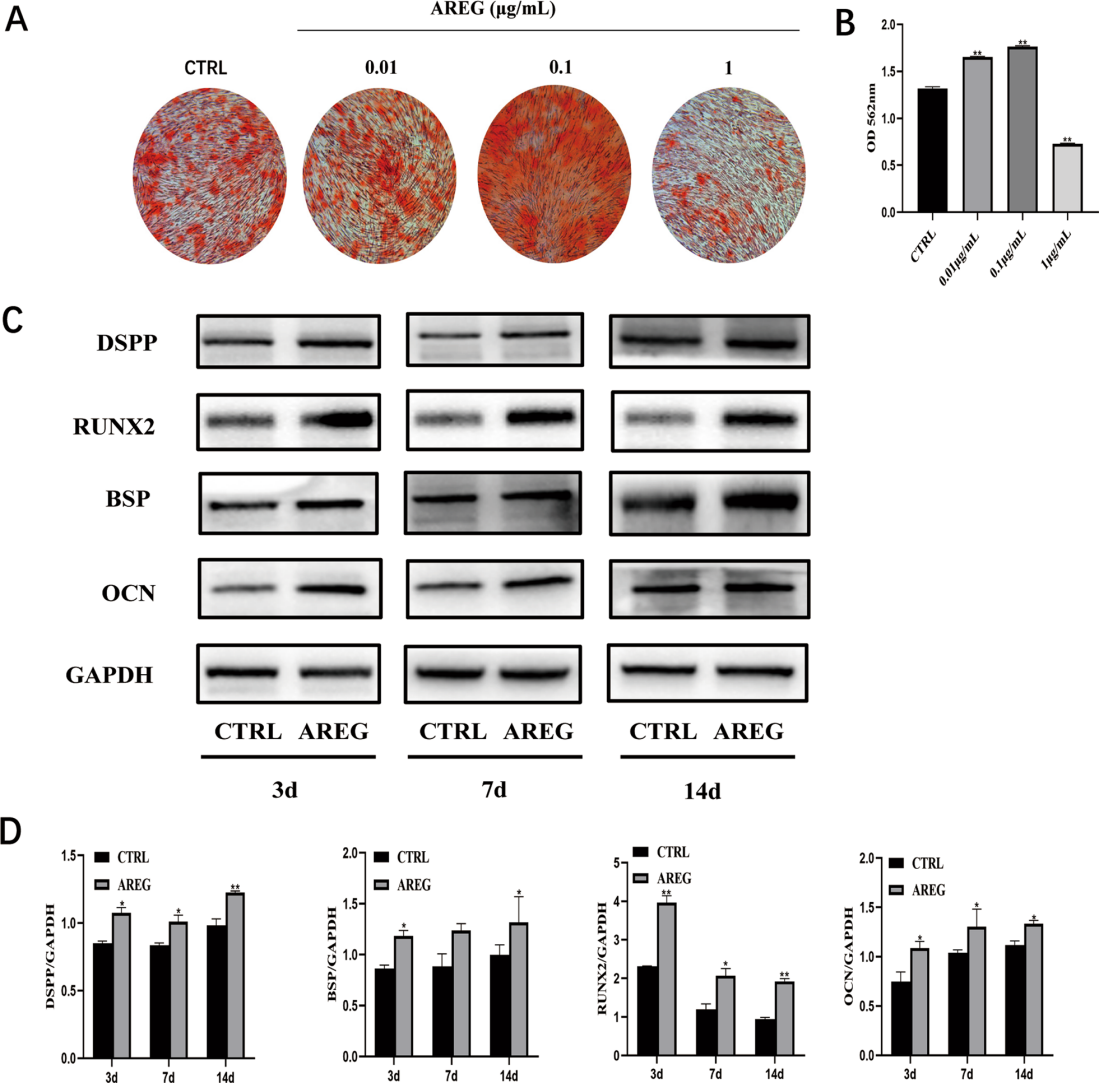
AREG enhances the oriented differentiation potential of DPSCs. **A** Alizarin Red S staining of DPSCs after 14 days’ culture with a range of AREG concentrations in mineralizing medium. **B** Quantitative analysis of Alizarin Red S staining (*n* = 5, **P < 0.05). **C** Western blot analysis showing expression of the odontoblastic markers DSPP, RUNX2, BSP, and OCN. The full-length gels and blots are included in Additional file 1: Fig. S1A. **D** ImageJ software analysis of the gray level of the panel. Data are expressed as means ± SDs, *n* = 3, *P < 0.05, **P < 0.01

After two weeks of osteo/odontogenic induction, it was apparent that AREG concentrations between 0.01 and 0.1 µg/mL promoted mineralized nodule formation in a dose-dependent manner, although a significant decrease was apparent at 1 µg/mL AREG exposure (Fig. 3A, B). Consequently, 0.1 µg/mL AREG exposure was selected as the optimal concentration for the following studies to investigate the influence of AREG on DPSCs. When the expression of odontoblastic marker proteins was examined by Western blotting, it was found that the levels of DSPP, BSP, RUNX2, and OCN were noticeably up-regulated in the AREG-treated groups compared with the controls by day 3. Levels were markedly elevated on days 7 and 14 (*P* < 0.05) (Fig. 3C, D). Taken together, these results indicated that AREG could stimulate DPSC differentiation.

### Effects of AREG on regeneration and mineralization of hDPSCs in vivo

**Fig. 4:**
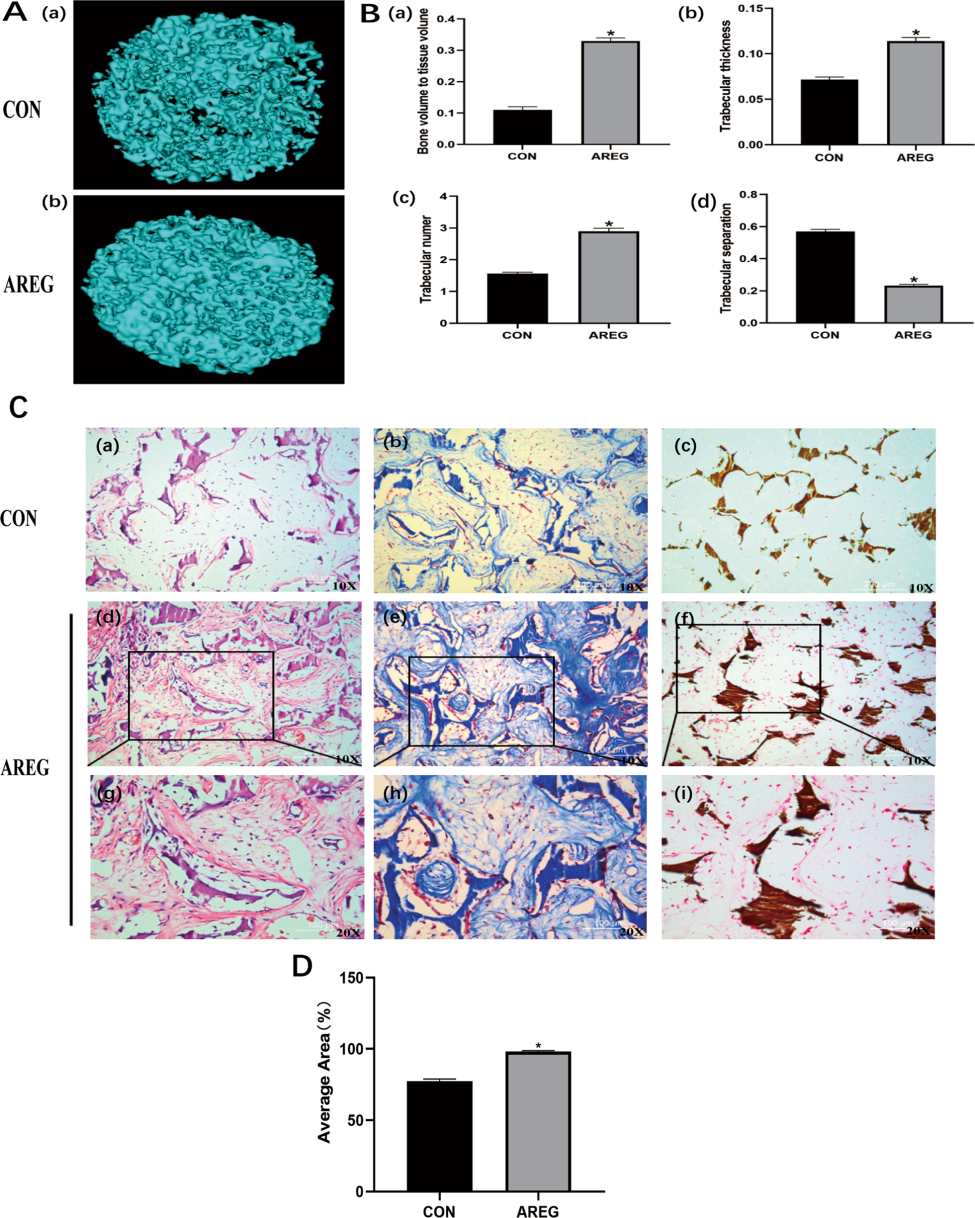
The effect of AREG on the regeneration and mineralization of hDPSCs in vivo*.* **A** Micro-CT images of the DPSC/S-scaffold construct in nude mice after subcutaneous implantation for 4 weeks. **A** (a) and (b) show the DPSC/NF-gelatin-scaffold composites of the control and AREG group, respectively. **B** Quantitative analysis of micro-CT images (a) BV/TV (Bone Volume to Tissue Volume); (b) Tb.Th. (Trabecular Thickness); (c) Tb.N. (Trabecular Number); (d) Tb.Sp. (Trabecular Separation). **C** Histological staining of the DPSC/S-scaffold construct after subcutaneous implantation in nude mice for 4 weeks. (a, d, g) H&E staining; (b, e, h) Masson staining; (c, f, i) Von Kossa staining. *P < 0.05 represents a significant change compared with the control

To analyze the regenerative capability in response to AREG, the cell-scaffold composites were subcutaneously implanted into nude mice. Subsequently, the specimens were scanned and images were reconstructed using micro-CT analysis (Fig. 4A, B). Micro-CT images showed the formation of more highly mineralized tissue in the 0.1 μg/mL AREG exposure group in the composites (Fig. 4A a, b). Additionally, quantitative analysis also indicated that the bone volume fraction, trabecular thickness, and trabecular number were superior in the AREG group compared with the control group, although the trabecular separation was decreased (Fig. 4B a–d). Combined, these findings supported the ability of AREG in enhancing the odonto/osteogenic potential of DPSCs.

The results of the H&E and Masson staining showed that the cell-scaffold composites stimulated by 0.1 μg/mL AREG produced greater DPSC penetration into the pores of scaffold, as well as showing greater extracellular matrix secretion and collagen fiber wrapping in the NF-gelatin scaffold, compared with the control group (Fig. 4C a, b, d, e, g, h). Von Kossa staining confirmed the presence of mineralization and further demonstrated greater deposition of mineralized matrix and calcium salts in the AREG exposure group (Fig. 4C c, f, i). These data confirmed that AREG promoted the regeneration and mineralization capability of hDPSCs in vivo.

### Effects of AREG overexpression and knockdown on hDPSCs differentiation

**Fig. 5:**
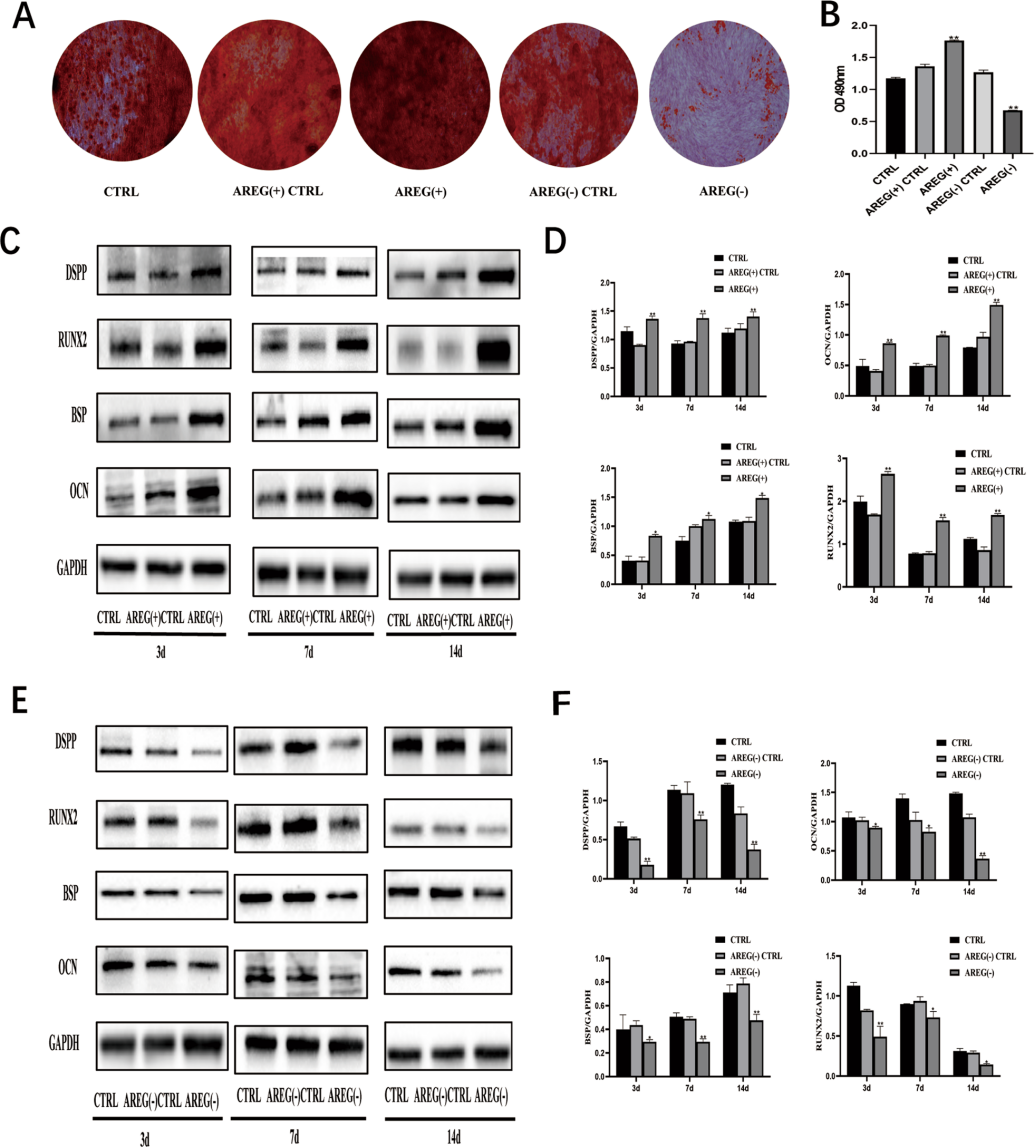
The effects of AREG overexpression/knockdown on the differentiation of DPSCs. **A** Alizarin Red S staining showing mineralized nodule formation in the AREG ( +) and AREG (–) groups. **B** Quantitative analysis of A (*n* = 5, **P < 0.05). **C** Protein expression of odontoblastic markers DSPP, RUNX2, BSP, and OCN shown by Western blotting in the AREG ( +) group. The full-length gels and blots are included in Additional file 1: Fig. S1B. **D** ImageJ analysis of the gray level of the panel. **E** Protein expression of odontoblastic markers DSPP, RUNX2, BSP, and OCN shown by Western blotting in the AREG ( −) group. The full-length gels and blots are included in Additional file 1: Fig. S1C. **F** ImageJ was used to analyze the gray level of the panel. Data represent means ± SDs, *n* = 3, *P < 0.05, **P < 0.01

To further investigate AREG function during DPSC differentiation, cells were transfected with the *AREG* vector to induce *AREG* overexpression. Western blotting demonstrated increased expression of the odontoblastic markers at 3, 7, and 14 days after induction, compared with the control group (Fig. 5C, D). Furthermore, overexpression of *AREG* promoted mineralized nodule formation in DPSCs as detected by ARS staining and quantitation (Fig. 5A, B). These data supported the ability of AREG to promote differentiation of DPSCs.

To investigate whether *AREG* up-regulation was necessary for DPSC differentiation, AREG expression was silenced by shRNA transfection in DPSCs. Silencing was confirmed by qRT-PCR at 72 h after transfection following puromycin selection. Protein expression of DPSC odontoblast markers was decreased in response to AREG inhibition, as shown by Western blotting (Fig. 5E, F). ARS staining and quantitation showed that AREG silencing attenuated mineralized nodule formation in DPSCs (Fig. 5A, B). These results indicated that reduced *AREG* expression resulted in the suppression of odontogenic differentiation.

### Involvement of MAPK signaling in AREG-induced differentiation of hDPSCs

**Fig. 6:**
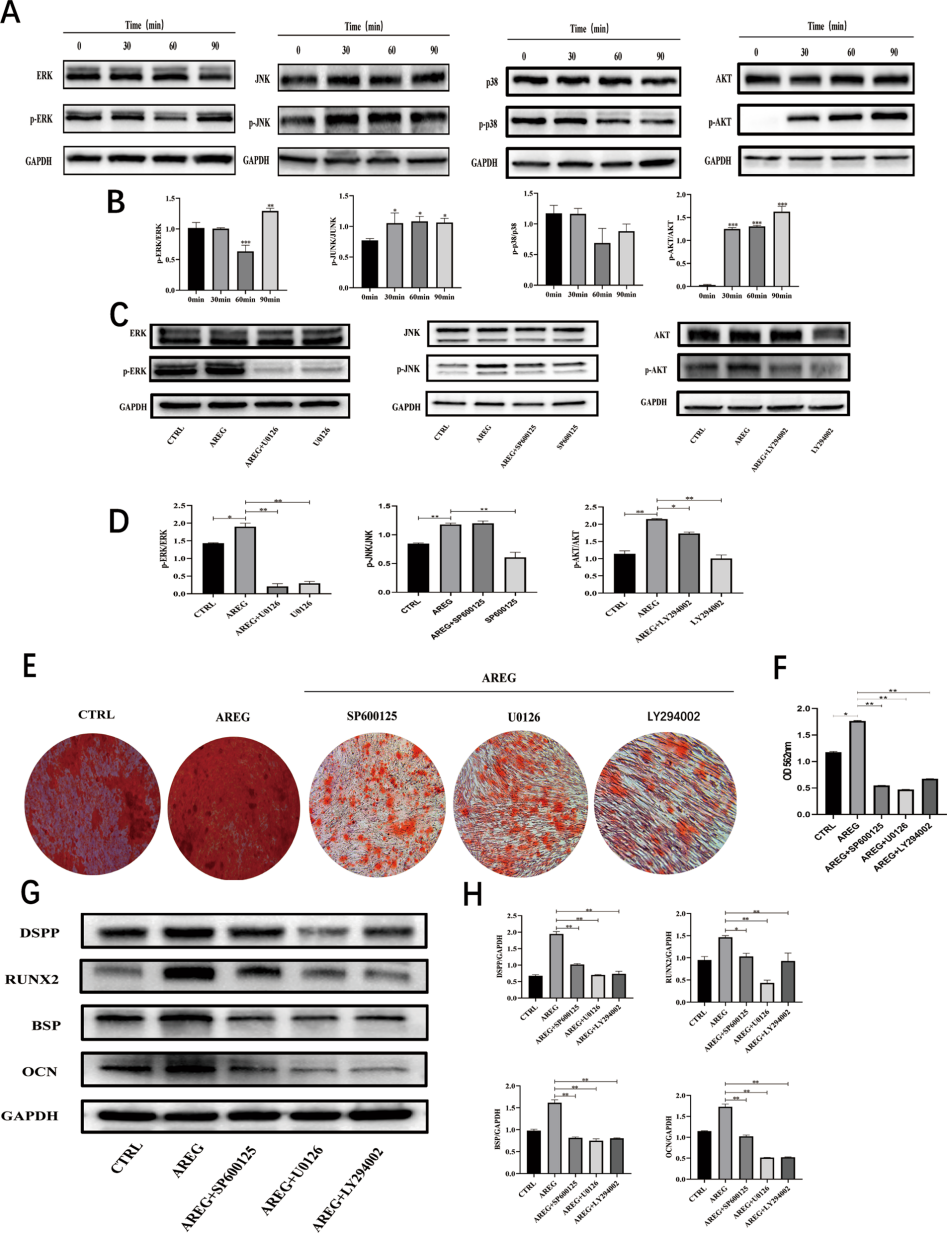
AREG promotes differentiation of DPSCs by activation of the ERK, JNK, and AKT pathways. **A** Protein levels of ERK and p-ERK, JNK and p-JNK, p38 and p-p38, and ATK and p-AKT treated with AREG at different time points (0, 30, 60, and 90 min) shown by Western blotting in DPSCs. The full-length gels and blots are included in Additional file 1: Fig. S2A. **B** Quantitative analysis of the gray intensity of A. **C** Treatment of DPSCs with specific ERK, JNK, or PI3K inhibitors (U0126, SP600125, and LY294002, respectively). Protein levels of ERK and p-ERK, JNK and p-JNK, p-AKT/AKT are shown by Western blotting at the indicated times. The full-length gels and blots are included in Additional file 1: Fig. S2B. **D** Quantitative analysis of the ratio of p-ERK/ERK, p-JNK/JNK and p-AKT/AKT from C. **E** Alizarin Red S staining showing mineralized nodule formation in DPSCs treated with the specific inhibitors. **F** Quantitative analysis of Alizarin Red S staining (*n* = 5, *P < 0.05). **G** Western blots showing protein expression of odontoblastic markers (DSPP, RUNX2, BSP, and OCN) in different groups at day 14. The full-length gels and blots are included in Additional file 1: Fig. S2C. **H** Quantitative analysis of data presented in G. Data represent means ± SDs, *n* = 3, *P < 0.05, **P < 0.01

Treatment with AREG increased the protein expression level of p-ERK after 60 min stimulation. AREG stimulation also resulted in phosphorylation of JNK in DPSCs (Fig. 6A, B). However, AREG showed minimal effects on phosphorylated p38 levels (Fig. 6A, B). Notably, incubation with the ERK, JNK, and p38 MAPK inhibitors markedly antagonized the effect of AREG on phosphorylated ERK and JNK in hDPSCs (Fig. 6C, D). These inhibitors also reduced the expression of the mineralization markers assayed by day 14, and mineralized nodule formation in DPSCs (Fig. 6E–H). Taken together, these data indicated that the ERK MAPK and JNK MAPK pathways are involved in AREG-induced differentiation of hDPSCs.

### Involvement of PI3K/AKT signaling in AREG-induced differentiation of hDPSCs

AREG phosphorylated AKT in DPSCs in a time-dependent manner (Fig. 6A, B); this effect was inhibited by the PI3K pathway inhibitor LY294002 (Fig. 6C, D). Furthermore, Western blotting showed that the levels of DSPP, BSP, RUNX2, and OCN were markedly reduced in cells treated with AREG + LY204002 (*P* < 0.05) (Fig. 6G, H). The PI3K pathway inhibitor LY294002 also markedly antagonized mineralized nodule formation and the expression of mineralization markers in DPSCs (Fig. 4E–H). These findings indicate that the PI3K/AKT pathways are involved in the AREG-induced differentiation of hDPSCs.

## Discussion

DPSCs provide a cellular reservoir which have significant applications in several tissue engineering applications. As with other somatic stem cells, a range of studies have demonstrated the potential of DPSCs in regenerative therapies, based on their multipotent differentiation capability [24]. Importantly, their cell growth and differentiation is regulated by action of several stimulatory growth factors and numerous studies have now demonstrated the importance of the EGF family in various development processes [25, 26]. EGF is known to exert its biological actions through its binding to EGFR on cell surfaces and AREG is also able to interact with the same receptor to activate downstream signaling pathways [10]. In the present study, results indicated that AREG was able to promote odontoblastic differentiation in hDPSCs through the activation of the ERK MAPK, JNK MAPK, and PI3K/Akt pathways, and facilitated the regeneration and mineralization of hDPSCs. This understanding now has the potential to be applied for the development of novel strategies for the repair and regeneration of damaged pulp tissue.

A previous study demonstrated that AREG was able to inhibit or induce cell growth and proliferation in a range of cell types [14]. Indeed, AREG was shown to induce a potent proliferative response in colon carcinoma cells [27] and an increased proliferation in airway epithelial and smooth muscle cells [28]. AREG plays a vital function in skin wound healing by stimulating keratinocyte proliferation [29–32], and it has been reported that overexpression of AREG induces self-sufficient growth and survival in lung, liver, colon, breast, and pancreatic carcinoma cells [10, 33–37]. In the present study, we observed that AREG exerted a biphasic effect. Data demonstrated that the numbers of hDPSCs treated with 0.01μg/mL and 0.1μg/mL AREG were significantly increased after 3, 5, and 7 days of treatment while exposure to the higher dose of 1μg/mL AREG reduced cell numbers by day 7. The analysis of cell cycle phases demonstrated that the 0.01 μg/mL and 0.1 μg/mL AREG treatment groups had a marginally higher proliferation index (PI = G2/M+S), in comparison with both the control group and the 1μg/mL AREG-treatment group. Notably, flow cytometry analysis showed that treatment with 0.01-1µg/ml AREG had minimal impact on apoptosis in hDPSCs. These results are largely consistent with those of current studies [29–32].

AREG is potentially suited for use in tissue repair and regeneration applications as it is not only able to enhance proliferation but can also stimulate differentiation. These data are consistent with previous findings as AREG has been reported to induce the differentiation of neuronal PC12 cells [38] and it has also been shown to be more effective for human mammary epithelial differentiation than other EGFR ligands [39]. Similar results have been reported for human mammary myoepithelial cells [40]. In our study, AREG was shown to induce mineralized nodule formation in a dose-dependent manner, although the high dose of 1 µg/mL AREG induced a marked decrease. In addition, AREG also increased DSPP, BSP, RUNX2, and OCN expression. Together, these data indicated that AREG induced odontogenic differentiation of DPSCs in vitro.

The biomimetic NF-gelatin scaffold used here provided an excellent resource for bone tissue engineering studies due to its physical architecture and chemical composition which is similar to that of natural bone ECM. Constructs exhibited excellent biocompatibility, mechanical stability, and enhancement of osteogenic differentiation [22, 23, 41]. To clarify the role of AREG in regeneration and mineralization in vivo, the DPSC/NF-gelatin-scaffold composites with or without AREG treatment were subcutaneously implanted in nude mice for 4 weeks. Micro-CT analysis was initially used to detect the content, density, and distribution of both bone tissue and mineralized hard tissue formed. The quantitative analysis using Micro CT showed that BV/TV (Bone Volume to Tissue Volume), Tb.Th. (Trabecular Thickness), and Tb.N. (Trabecular Number) were markedly increased in the AREG-stimulated group, although the trabecular separation was decreased. These findings indicate that AREG supplementation produced more newly mineralized tissue together with increased numbers and thicker bone trabeculae, and that the trabecular structure was more compact. Importantly, AREG supplementation also promoted the formation of the mineralized tissue in vivo. Furthermore, both H&E and Masson staining showed increased DPSC penetration into the pores of the scaffold, with relatively large amounts of ECM and collagen fiber deposition and formation in the AREG group. Von Kossa staining indicated that biomineralization occurred in all the composites, with greater mineralized matrix and calcium salt deposition on the composites in the AREG treated group. This finding was consistent with the micro CT quantitative analysis data. All the results demonstrated that AREG facilitated the formation of a dentin-like matrix in DPSC/NF-gelatin-scaffold composites. Furthermore, AREG overexpression experiments showed a similar promotion of odontogenic differentiation, whereas the knockdown experiments inhibited this process. These results indicated that AREG was necessary for odontoblastic differentiation of DPSCs and could facilitate the regeneration and mineralization of hDPSCs. Although not yet determined, it is possible that AREG may contribute substantially to injured pulp repair and regeneration.

AREG plays a vital role in biological processes through its interaction with EGFR and tyrosine phosphorylation of downstream proteins, activating the two major intracellular pathways of MAPK and PI3K/Akt signaling [42, 43]. Interestingly, a previous study has shown that AREG interacts with EGFR, activating PI3K/Akt, and subsequently inducing the NF-κB transcription factor interaction with the MMP-13 promoter, inducing cartilage destruction in osteoarthritis [44]. MAPK signaling has also been observed to participate in AREG-induced morphological effects in MDCK cells [45]. Indeed, AREG stimulation was found to be required for the differentiation of K5 + K19-hMECs through activation of ERK and MAPK but not Akt signaling [39]. Moreover, inhibition of ERK1/2 blocked AREG-induced myoepithelial differentiation [40]. In the present study, ERK MAPK, JNK MAPK, and AKT were phosphorylated in response to AREG stimulation, an action that was markedly antagonized by specific inhibitors of these proteins. In addition, the ERK MAPK, JNK MAPK, and PI3K/Akt inhibitors also significantly reduced mineralized nodule formation and expression of protein mineralization markers. These data support the involvement of ERK MAPK, JNK MAPK, and PI3K/AKT pathways in the AREG-induced differentiation of hDPSCs.

## Conclusions

In conclusion, our in vitro and in vivo data demonstrated that AREG was necessary for odontoblastic differentiation of DPSCs and promoted their regeneration and mineralization potential. Furthermore, the investigation of the roles of the MAPK and PI3K/AKT pathways in AREG-induced growth and osteo/odontogenic differentiation in hDPSCs implicated ERK MAPK, JNK MAPK, and PI3K/AKT signaling in AREG-mediated differentiation of hDPSCs. Further investigation of these mechanisms may provide novel targets and treatment modalities for use in the repair of damaged and diseased pulp and the regeneration of dental tissues.

## Supplementary Information

**Additional file 1: Supplementary Figure 3**. The picture shows the full length of the strip, and the strips circled in the red box are the strips used in the article. A corresponds to Figure C in Fig 3. B corresponds to Figure C in Fig 5. C corresponds to Figure E in Fig 5. **Supplementary Figure 5**. The picture shows the full length of the strip, and the strips circled in the red box are the strips used in the article. A corresponds to Figure A in Fig 6. B corresponds to Figure C in Fig 6. C corresponds to Figure G in Fig 6.

## Data Availability

The authors confirm the availability of all data generated or analyzed in this manuscript.

## Abbreviations

AREG: Amphiregulin
MAPK: Mitogen-activated protein kinase
PI3K: Phosphatidylinositol 3-kinase
ERK: Extracellular signal-regulated kinase
JNK: C-Jun N-terminal kinase
EGF: Epidermal growth factor
DSPP: Dentin sialophosphoprotein
RUNX2: Runt-related transcription factor 2
OCN: Osteocalcin
BSP: Bone sialoprotein
NF-gelatin: Nanofibrous gelatin
BV/TV: Bone volume to tissue volume
Tb.Th.: Trabecular thickness
Tb.N.: Trabecular number
Tb.Sp.: Trabecular separation
