# Supplementary material for: Amphiregulin regulates odontogenic differentiation of dental pulp stem cells by activation of mitogen-activated protein kinase and the phosphatidylinositol 3-kinase signaling pathways
Source: Stem Cell Res Ther. 2022 Jul 15;13:304. doi: 10.1186/s13287-022-02971-4 (PMC9284861; doi:10.1186/s13287-022-02971-4)
Supplement: Supplementary file 1 — Additional file 1: Supplementary Figure 3. The picture shows the full length of the strip, and the strips circled in the red box are the strips used in the article. A corresponds to Figure C in Fig 3. B corresponds to Figure C in Fig 5. C corresponds to Figure E in Fig 5. Supplementary Figure 5. The picture shows the full length of the strip, and the strips circled in the red box are the strips used in the article. A corresponds to Figure A in Fig 6. B corresponds to Figure C in Fig 6. C corresponds to Figure G in Fig 6. [file 13287_2022_2971_MOESM1_ESM.docx]

**Amphiregulin regulates differentiation of dental pulp stem cells by activation of mitogen-activated protein kinase and the phosphatidylinositol 3-kinase signaling pathways**

*Junqing Li, DDS, MS,^1, 2a^ Zhihua Wang, DDS, MS, ^1a^ Juan Wang, DDS, MS,^1a^ Qian Guo, DDS, MS,^1^ Yi Fu, DDS, MS,^1, 2^ Zihan Dai, DDS, MS,^1^MinghaoWang, DDS, PhD,^1^ Yu Bai, DDS, PhD, ^1^ Xin Liu, DDS, MS,^1^ Paul Cooper, DDS, PhD,^2^ Jiayuan Wu, DDS, PhD,^3^* and Wenxi He, DDS, PhD^4^*.*

**A.**


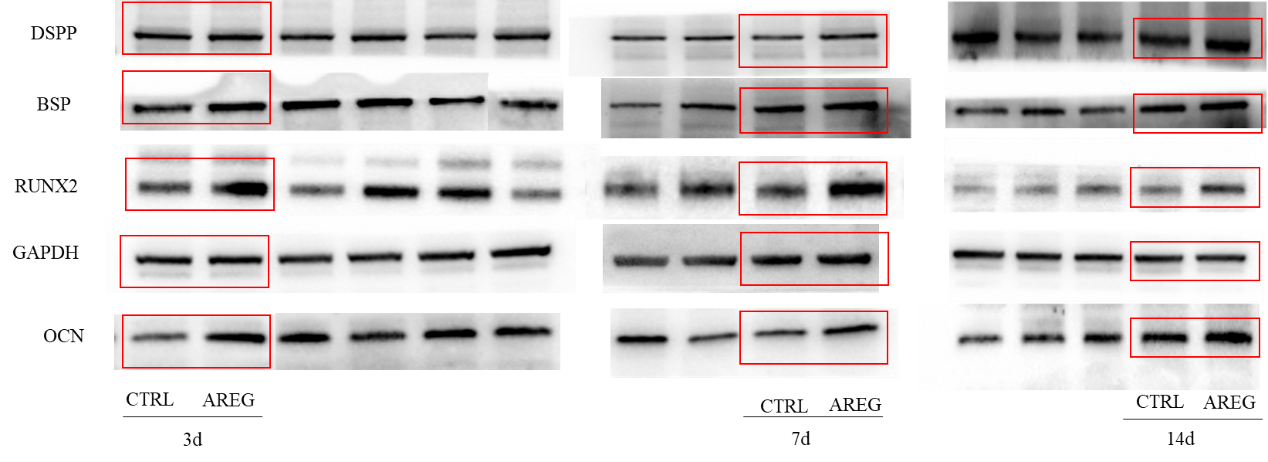


**B.**


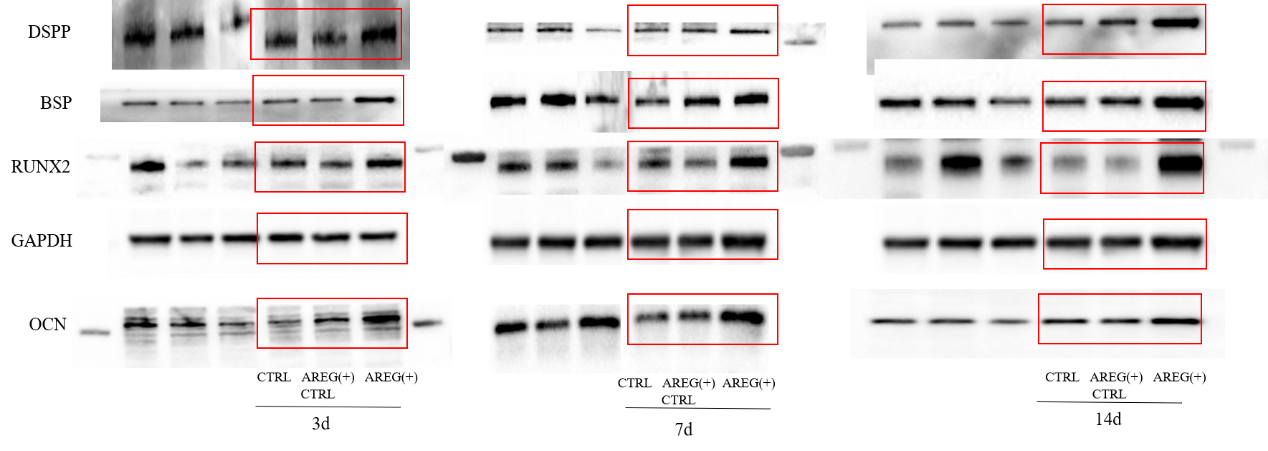


**C.**


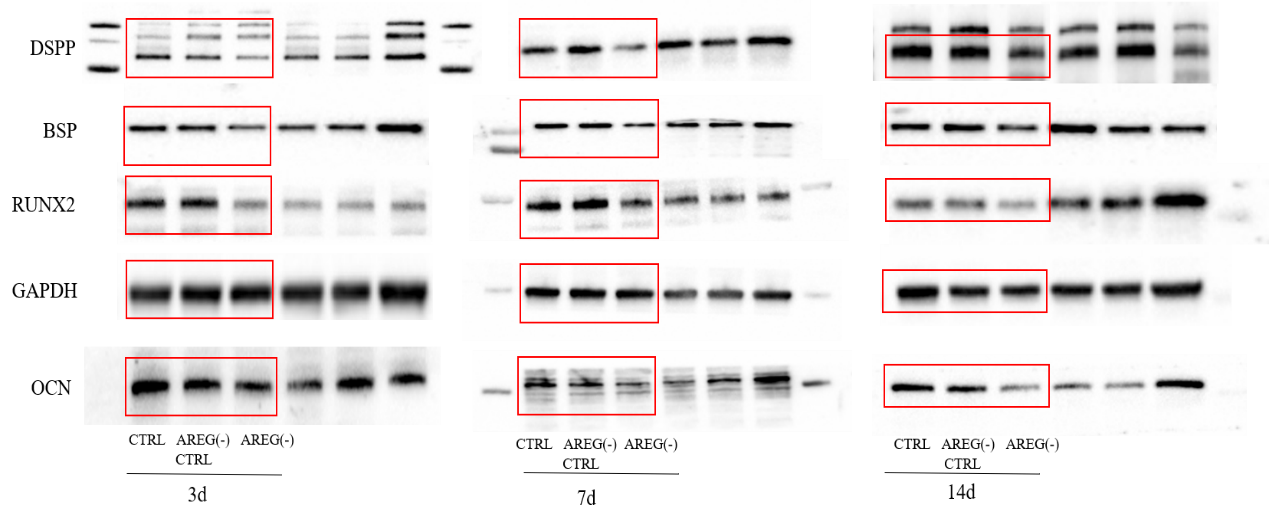


Supplementary Figure 3 and 5.

The picture shows the full length of the strip, and the strips circled in the red box are the strips used in the article. A corresponds to Figure C in Fig 3. B corresponds to Figure C in Fig 5. C corresponds to Figure E in Fig 5.

**A.**


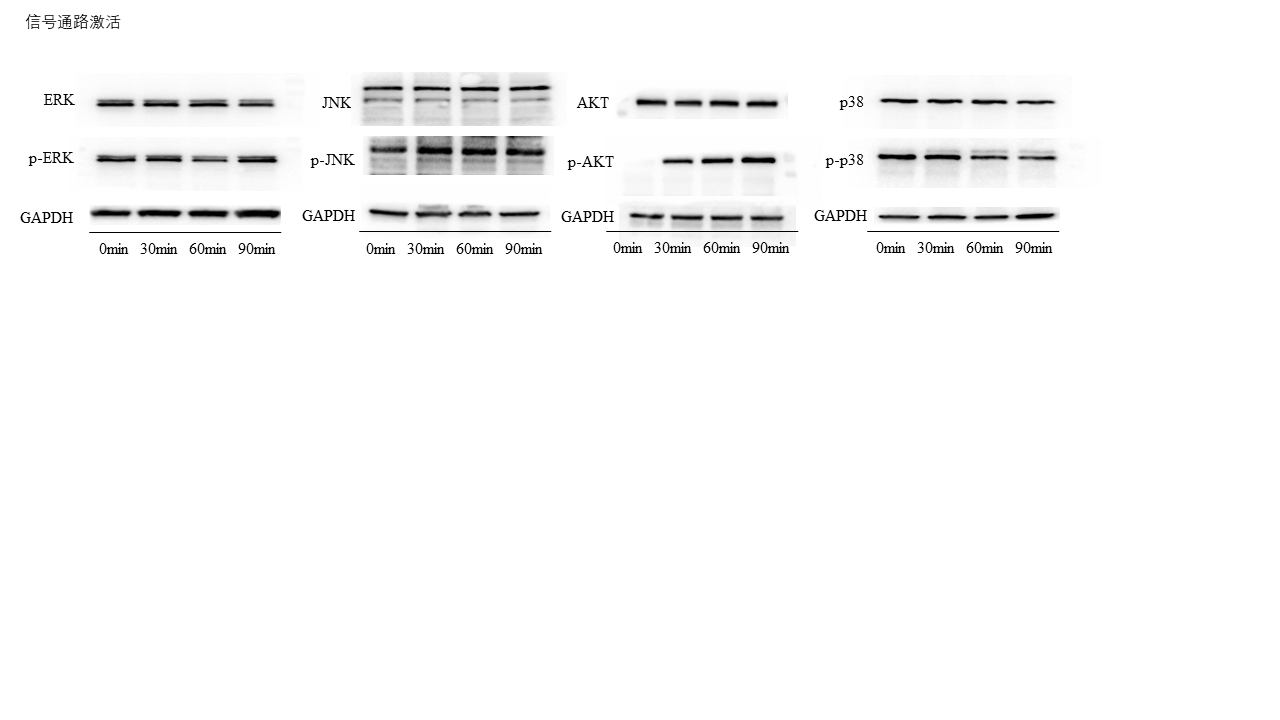


**B.**


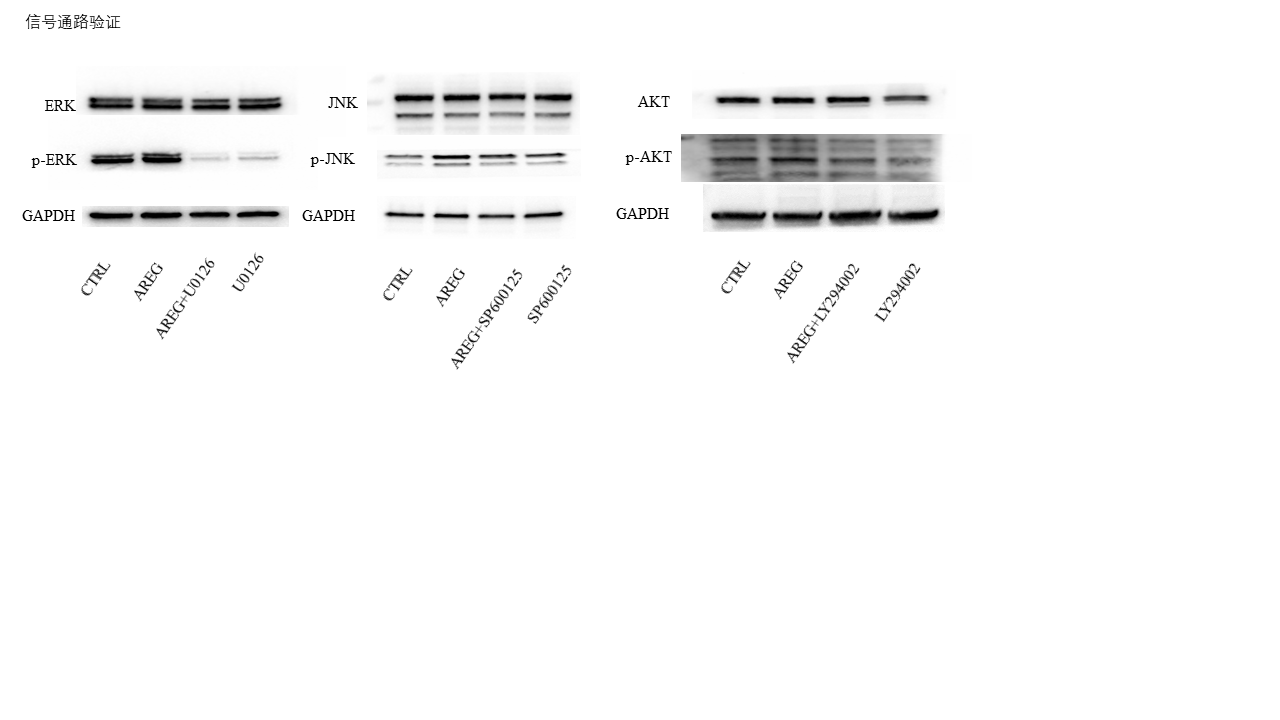


**C.**


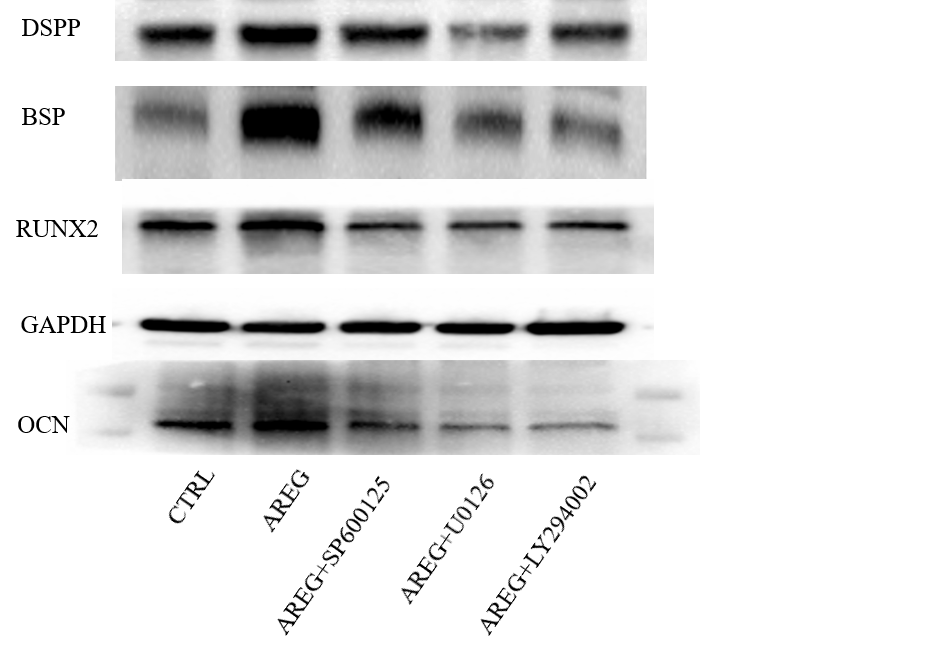


Supplementary Figure 5.

The picture shows the full length of the strip, and the strips circled in the red box are the strips used in the article. A corresponds to Figure A in Fig 6. B corresponds to Figure C in Fig 6. C corresponds to Figure G in Fig 6.
